# Thirty-five years of research into ribozymes and nucleic acid catalysis: where do we stand today?

**DOI:** 10.12688/f1000research.8601.1

**Published:** 2016-06-27

**Authors:** Sabine Müller, Bettina Appel, Darko Balke, Robert Hieronymus, Claudia Nübel

**Affiliations:** 1 Institute of Biochemistry, Ernst-Moritz-Arndt University Greifswald, Greifswald, Germany

**Keywords:** ribozyme, catalytic, RNA

## Abstract

Since the discovery of the first catalytic RNA in 1981, the field of ribozyme research has developed from the discovery of catalytic RNA motifs in nature and the elucidation of their structures and catalytic mechanisms, into a field of engineering and design towards application in diagnostics, molecular biology and medicine. Owing to the development of powerful protocols for selection of nucleic acid catalysts with a desired functionality from random libraries, the spectrum of nucleic acid supported reactions has greatly enlarged, and importantly, ribozymes have been accompanied by DNAzymes. Current areas of research are the engineering of allosteric ribozymes for artificial regulation of gene expression, the design of ribozymes and DNAzymes for medicinal and environmental diagnostics, and the demonstration of RNA world relevant ribozyme activities. In addition, new catalytic motifs or novel genomic locations of known motifs continue to be discovered in all branches of life by the help of high-throughput bioinformatic approaches. Understanding the biological role of the catalytic RNA motifs widely distributed in diverse genetic contexts belongs to the big challenges of future RNA research.

## Introduction

Nowadays, the term 'ribozyme' to designate an RNA catalyst is used with the same implicitness as the term ‘enzyme’ has always been used for proteinaceous biocatalysts. The fact that RNA can cleave and ligate itself, that cleavage of the 5′-trailer of tRNA in tRNA processing is mediated by the RNA subunit of RNase P, that introns may undergo self-splicing, and that the spliceosome and, even more impressively, the ribosome are actually ribozymes meanwhile has found entry into the textbooks. The exciting field of research into RNA catalysis started more than 30 years ago and over the first two decades was dominated by the discovery and identification of several classes of ribozymes occurring in nature and the elucidation of their catalytic structures and mechanisms. Apart from the ribosome that catalyses the formation of the peptide bond, all ribozymes discovered so far in nature support cleavage or ligation of a phosphodiester bond or both. However, the powerful method of SELEX (systematic evolution of ligands by exponential enrichment), originally developed for the selection of high-affinity RNA binders (aptamers) from a random library ^1, 2^, was adapted to the selection of ribozymes (and, moreover, DNAzymes) to catalyse a broad range of reactions, thus greatly enhancing the spectrum of nucleic acid catalysis ^3, 4^. Over the years, an enormous amount of data were obtained on high-resolution structures and the mechanisms of ribozymes ^5, 6^. All of this contributed to an understanding of ribozyme catalysis to an extent that has allowed engineering of ribozymes and DNAzymes with pre-determined functionality. Thus, the past decade has seen impressive developments based on the usage of known catalytic motifs in ribozyme-based switches for therapeutic and environmental diagnostics ^7^ and more recently for control of gene expression ^8^. In parallel, the ability of RNA to catalyse a wide variety of chemical reactions has revitalised the RNA world hypothesis, a postulated period in the origin of life in which RNA was the main player, for one as carrier of genetic information and for the other as catalyst ^9^. Early life may have started with self-replicating RNA, and a great deal of effort has been invested in developing ribozymes capable of self-replication ^10^ or, even more challenging, of catalysing RNA polymerisation ^11, 12^. The interest in RNA-world-relevant ribozyme activities continues, and one may well expect that there will be more to come.

In addition to ribozyme-based applications and RNA world scenarios as central topics of current research in the field, the frequency of ribozymes in nature and their function are of ongoing interest. With the help of high-throughput bioinformatic approaches, new ribozymes or novel genomic locations of known catalytic RNA motifs in highly diverse genetic contexts have been discovered in all branches of life ^13– 16^, and current research addresses the question of their biological role.

The field of ribozyme research has changed the focus from discovery and mechanistic/structural characterisation of ribozymes towards functional engineering into application. Nevertheless, the excitement of the first days of ribozyme discovery has carried over throughout the years; the search for new ribozymes or just ribozyme locations continues in all kingdoms of life—in particular, in the human genome. Moreover, as mentioned above, the search for RNA-world-relevant ribozyme activities continues with unchanged curiosity. A number of excellent review articles have summarised the achievements in nucleic acid catalysis (for recent examples, see 5, 17– 19). Here, we will concentrate on recent discoveries and developments in the field to draw a concise picture of ribozyme research and RNA and DNA catalysis 35 years after its beginning.

## Ribozyme-based switches

Over the past decade, it has become increasingly clear that the conformational flexibility of RNA is an important determinant of cellular function. In this regard, riboswitches located in the 5′-untranslated region (5′-UTR) of specific mRNAs have gained much attention ^20^. Composed of an aptamer and an expression platform, riboswitches regulate, in a ligand-dependent manner, gene expression at the level of transcription or, alternatively, translation. Binding of a specific ligand to the aptamer induces a conformational change in the expression platform, turning gene expression ON or OFF. Interestingly, this principle of allosteric regulation was used in the test tube before it was discovered in nature ^21^. By combination of ribozymes with aptamers, ribozyme activity was rendered ligand dependent and, consequently, adjustable. RNA or DNA aptamers for binding to a desired molecule can be produced by SELEX and linked to the ribozyme via a communication module, a sequence that translates the binding event occurring in the aptamer unit into an activity-associated conformational change within the ribozyme part. Thus, ribozyme activity can be used as a readout for a binding event, which in the case of a multiple turnover reaction would even lead to signal amplification. Owing to their modular composition of DNAzyme or ribozyme and aptamer, such constructs were termed aptazymes ^22^. Beyond the significance of aptazymes for medicinal diagnostics and therapy, RNA- and especially DNAzyme-based biosensors have gained importance as tools in environmental monitoring, in particular to detect environmental pollutants, such as toxic heavy metals, air- and water-borne microbes, and other toxins ^23^.

**Figure 1.**
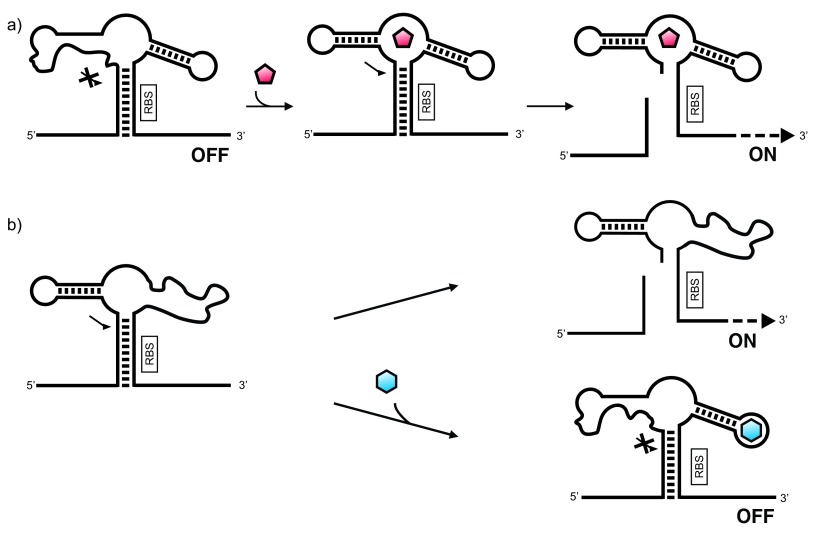
Ribozyme-based ON ( **a**) and OFF ( **b**) switches. The ribozyme-based device is positioned in the 5′-untranslated region (5′-UTR) of the transcript of interest. ( **a**) In the absence of a specific ligand, the ribozyme is inactive and the ribosome-binding site (RBS) is sequestered in a double-stranded region; translation is switched OFF. Upon ligand binding, the ribozyme is activated and cleavage can take place. As a result, the RBS is set free and translation can proceed. ( **b**) In the absence of a specific ligand, the ribozyme undergoes self-cleavage, thereby freeing the RBS and allowing translation to proceed. Binding of the ligand inhibits ribozyme activity, and translation is switched OFF.

Allosteric regulation of ribozyme activity has been used in a variety of contexts in the life sciences. Here, significant effort has been made in artificially modulating gene expression by a chemical signal. A ribozyme-based device positioned in the 5′- or 3′-UTR of a transcript and acting as a regulatory unit is partitioned between two functional conformations: one representing a ribozyme active state, the other an inactive state ^24, 25^. Ligand binding would support one of the two states, dependent on the specific design. As a consequence of ligand binding, translation is switched ON or OFF ( Figure 1). The advantage is that the effector molecule (ligand) binds directly to the regulatory module, without the involvement of proteins, such as transcription factors, which usually mediate genetic control. After some pioneering work in the early 2000s, important progress has been made in engineering ligand-dependent ribozyme modules that switch expression of suitable reporter genes, often using the hammerhead ribozyme to control stability of the target transcript ^26^. Furthermore, the genomic hepatitis delta virus ribozyme was engineered to control gene expression in mammalian cells and, when placed in tandem configuration, to construct a NOR logic gate device, demonstrating the modular composition of ribozyme-based RNA devices ^27^. Moreover, the recently discovered twister ribozyme, a highly flexible and active endonucleolytic ribozyme, has been used for the development of genetic switches ^28^. In all of these approaches, stability of a target transcript is modulated through conditional control of the cleavage activity of a ribozyme conjugated with a naturally occurring or *in vitro* selected aptamer domain and placed at a suitable position of the transcript. Ribozyme-based genetic control has been performed in different organisms ^27, 29, 30^ and in response to diverse ligands ^26, 30– 32^. In addition to chemical signals (ligand-responsive switches), physical signals (light or temperature) can be used to control ribozyme activity in such devices ^33^. Beyond regulation of bacterial or mammalian genes, the potential of ribozyme-based genetic switches for regulation of DNA and RNA viruses has been demonstrated ^34^. In particular, genome replication, infectious particle production and cytotoxicity of adenoviruses, and (in the case of a measles virus) progeny infectivity and virus spread were reduced by aptazyme-mediated control of gene expression, paving the way for future applications in medicine and virology.

Important challenges in the engineering of ribozyme-based switches by modular composition are the link between ribozyme (actuator) and ligand-responsive aptamer (sensor) and the relatively slow kinetics of secondary structure changes induced by ligand binding, thus limiting the regulatory potential of ribozyme-based switches ^24, 35^. Therefore, it is all the more important that powerful protocols for *in vivo* selection and screening and for high-throughput cellular RNA device engineering have been developed ^24– 26, 28, 36^. In general ribozyme-based switches allow for the regulation of gene expression by up to 30-fold ^26, 27^. However, it can be anticipated that, based on novel protocols for RNA device engineering and on the ever-growing understanding of the underlying structure-function relationships, novel designs will outperform those currently available.

## DNAzymes

**Figure 2.**
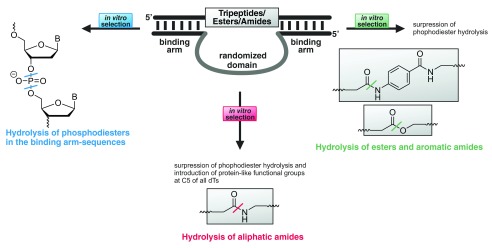
Selection of a DNAzyme from a random library under varying conditions. For more detail, see the ‘DNAzymes’ section of the main text.

As mentioned above, protocols for *in vitro* selection of nucleic acid catalysts from random libraries have paved the way for the development of artificial RNAzymes and DNAzymes. One of the most proficient DNAzymes, the so-called 10–23 motif, was selected back in 1997 ^37^ and was fully characterised in 1998 ^38^ and since then has been used as a scaffold in a large number of re-selections and rational designs. In addition, novel DNAzymes were selected from fully randomised libraries. The chemical repertoire of DNAzymes is surprisingly broad, ranging from cleavage of phosphodiester, ester, and amide bonds over supporting C-C bond-forming reactions up to the repair of thymine dimers, peptide modifications, and others (excellently reviewed in 19). The recently achieved DNA-catalysed amide hydrolysis ^39^ is a good example of the challenges in DNAzyme development. Previous selection experiments had led to DNA-catalysed DNA phosphodiester cleavage instead of the desired amide hydrolysis ^40^, and, under conditions that deliberately avoided phosphodiester hydrolysis, no DNAzyme with activity for hydrolysis of an aliphatic amide bond was found. Instead, selection resulted in DNA catalysts that supported hydrolysis of carbonic acid esters or of aromatic amide bonds ^41^. Only the inclusion, in the selection experiment, of nucleotide derivatives with attached protein-like functional groups allowed the identification of DNAzymes capable of aliphatic amide hydrolysis ( Figure 2) ^39^.

**Figure 3.**
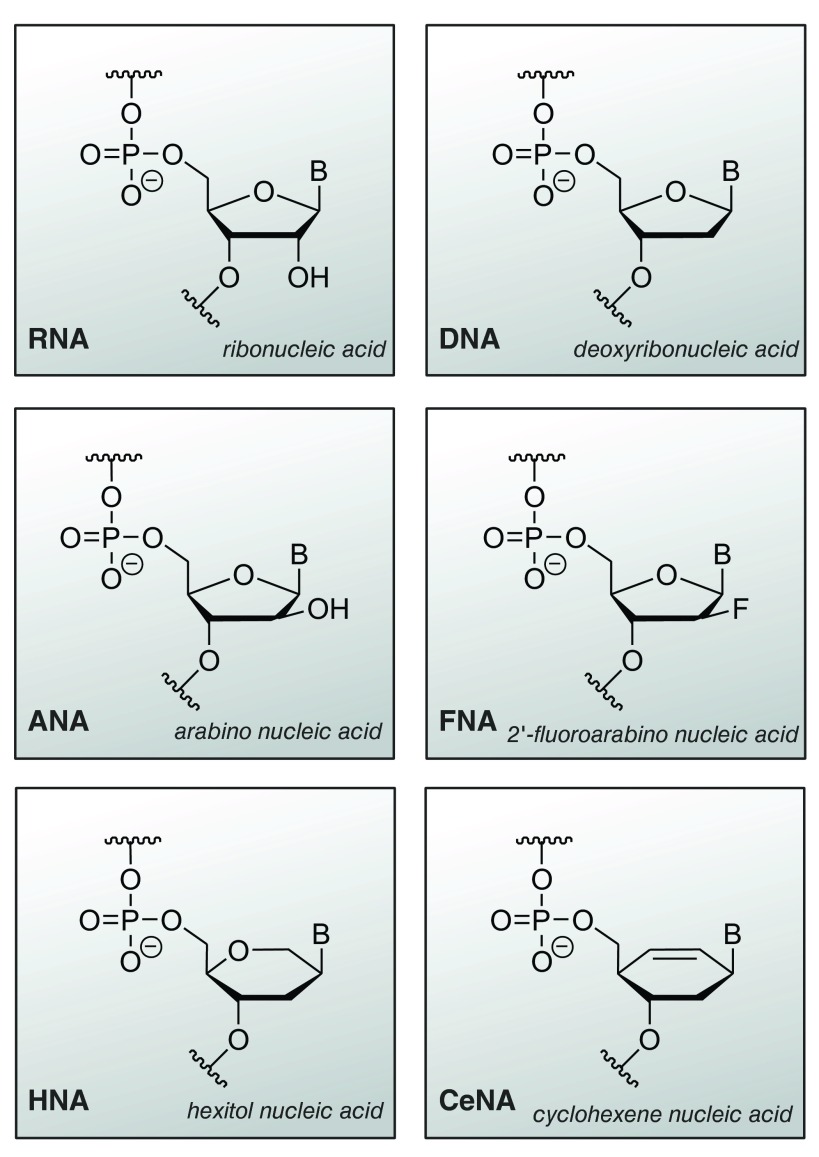
Structure of xeno nucleic acids in comparison with DNA and RNA. ANA, arabino nucleic acid; CeNA, cyclohexene nucleic acid; FANA, 2′-fluoroarabino nucleic acid; HNA, hexitol nucleic acid.

There has also been some effort in elucidating the structure of DNA catalysts. A recent breakthrough is the crystal structure of an RNA-ligating deoxyribozyme at 2.8 Å resolution ^42^. The structure gives new insight into the principles underlying DNA catalysis and allows conclusions to be drawn on the similarities and differences between RNAzymes and DNAzymes. Notably, the structure revealed that DNA can explore a wide range of conformations owing to a less restrictive sugar puckering as compared with RNA, and this feature compensates for the lack of the 2′-OH group that is present and structurally important in RNA ( Figure 3).

In general, DNAzymes continue to be developed as functional modules in biosensors and computing circuits ^23, 43– 47^ as well as for therapeutic use ^48^. For example, recent progress was made in the development of variants of the 10–23 DNAzyme against hepatitis C virus ^49^ and for the treatment of basal cell carcinoma ^50^ as well as in DNAzyme-mediated modification of allergen-induced asthmatic responses ^51^.

## XNAzymes

Very recently, an exciting new class of nucleic acid catalysts has emerged. Artificial endonuclease and ligase enzymes composed of synthetic genetic polymers, xeno nucleic acids (XNAzymes), were selected from random libraries in a method termed ‘cross-chemistry selective enrichment by exponential amplification’ (X-SELEX) ^52, 53^. As an essential prerequisite of the experiments, a modified DNA polymerase was engineered to tolerate the XNA building blocks (triphosphates) for polymerisation ^54^. Four different XNAs ( Figure 3) were used in the selection: arabino nucleic acids (ANAs), 2′-fluoroarabino nucleic acids (FANAs), hexitol nucleic acids (HNAs), and cyclohexene nucleic acids (CeNAs), and for all of them catalytically active species were found after 10 to 20 rounds of selection ^52^. Moreover, a FANA metalloenzyme with activity for ligation of FANA was identified, thus establishing catalysis in an entirely synthetic system ^53^. These results have strong implications for the emergence of life on earth, underscoring the possibility that genetic polymers with backbones other than ribose may have pre-dated the emergence of RNA and the RNA world.

## Ribozymes in RNA world scenarios

**Figure 4.**
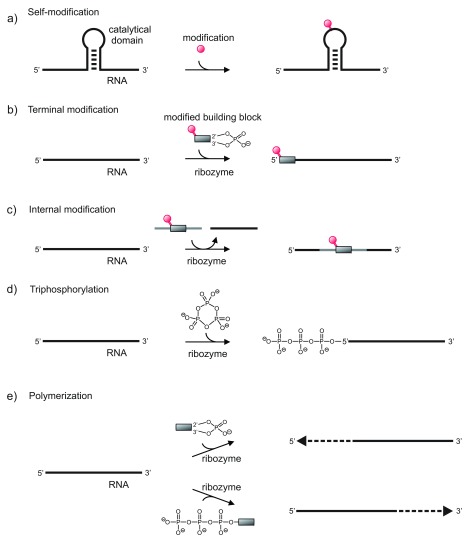
Schematic presentation of ribozyme activities that might have played a role in the RNA world. **a**) self-modification, e.g. alkylation; **b**) 5'-terminal modification by ribozyme-supported addition of an activated building block; **c**) internal modification by ribozyme-supported fragment exchange; **d**) ribozyme-supported 5' –triphosphorylation with trimetaphosphate; **e**) ribozyme-supported RNA polymerization with nucleoside-2',3'-cyclic phosphates (in 3'→5'-direction) or nucleoside-5'-triphosphates (in 5'→3'-direction) as activated building blocks.

The discovery of ribozymes has led to a renaissance of the RNA world theory, and ever since much effort has been put into the identification of ribozymes with useful activities in a time period when life was based on RNA functioning as both genome and genome-encoded catalyst ^9^. Thus, a number of *in vitro* selections aimed at the identification of RNA catalysts supporting reactions that might have been used by RNA world organisms were carried out. The synthesis of RNA certainly would have been a core activity, and ribozymes for reaction steps involved in RNA synthesis have been generated ^55^. Recent success has been made in ribozyme-mediated triphosphorylation of RNA-5′-hydroxyl groups using cyclic trimethaphosphate as the energy source ^56, 57^, in ribozyme-mediated self-replication ^10, 58^, and in polymerisation of activated nucleotides ^11, 12, 59^. In addition, other recently demonstrated activities, such as ribozyme-mediated RNA processing ^60, 61^, recombination ^62, 63^, nucleotide addition ^64^, and self-alkylation ^65^ (some of them illustrated in Figure 4), speak to the capacity of RNA to support a wide variety of reactions with relevance in RNA world scenarios.

## New catalytic motifs

**Figure 5.**
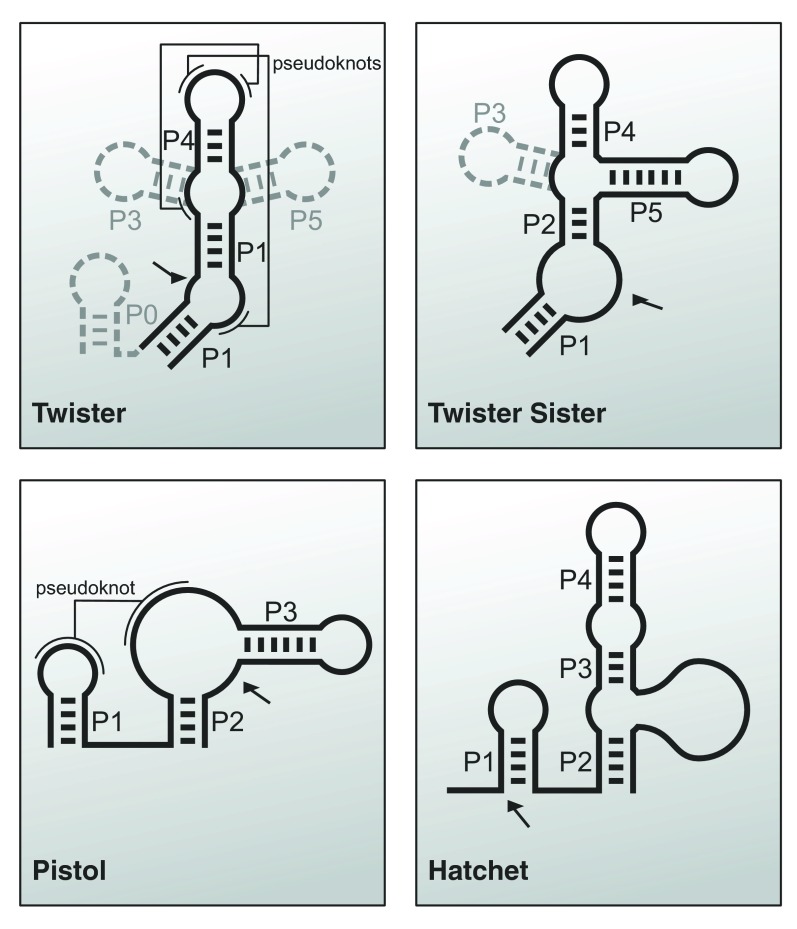
Secondary structures of recently discovered ribozymes. The arrows denote the cleavage sites.

Until recently, 10 classes of ribozymes existing among contemporary organisms were known, the hammerhead and hairpin ribozyme probably being the most prominent examples. The years after the discovery of these ribozymes were filled with investigations into their structures and catalytic mechanisms, and it took a rather long time until the question for additional naturally occurring ribozymes was addressed. The first of the recently discovered self-cleaving RNAs constituting the eleventh class of ribozymes is a small catalytic RNA motif, present in many species of bacteria and eukaryotes ^15^. In keeping with the tradition of giving ribozymes names related to their secondary structure, the new motif was called twister because of its small yet complex consensus structure composed of three stems conjoined by internal and terminal loops and a two-pseudoknot tertiary fold ( Figure 5) ^66– 68^. With an *in vivo* cleavage rate of 1000 per minute, the twister ribozyme is one of the fastest self-cleaving ribozymes, and based on biochemical experiments in conjunction with molecular dynamics simulation, a mechanism involving general acid-base catalysis by a conserved active site adenine residue has been proposed ^69^. This is in general agreement with the mechanisms of other self-cleaving ribozymes like the hairpin or the hepatitis delta virus ribozyme, which also require an adenine residue in the active site ^5^. However, apparently there is a striking difference: whereas in the hairpin and hepatitis delta virus ribozyme, N1 of adenine is involved in catalysis, N3 of adenine was suggested as a strong candidate to act as general base in twister ribozyme-mediated self-cleavage ^69^. This is particularly interesting because, if indeed N3 takes this role, it would expand the mechanistic repertoire of the small endonucleolytic ribozymes.

High-throughput bioinformatics assisted the identification of additional self-cleaving candidates named twister sister, pistol, and hatchet ribozyme ^70^, which upon *in vitro* characterisation were shown to indeed be ribozymes ^70– 72^. All of these new ribozymes support a transesterification reaction yielding a 5′-hydroxyl group and a 2′,3′-cyclic phosphate at the cleavage site. A recent review of the chemistry and biology of self-cleaving ribozymes referring also to the four new ribozyme classes can be found in 5.

## Future prospects

Over the years, ribozyme research and nucleic acid catalysis have remained a very exciting field with unchanged potential for new discoveries. A strong focus of current research is the uncovering and understanding of the role that ribozymes play in biological systems. The first results on the influence of self-cleaving RNA structures on genetic control are just emerging. For example, the hammerhead, the hepatitis delta virus-like, and the twister ribozyme are widespread in nature and appear in rather diverse genetic contexts ^14, 15, 73^. Ribozymes have been identified in intronic regions and mobile genetic elements, suggesting a role in pre-RNA and transcript processing ^13, 74, 75^. Understanding this additional level of genetic control and regulation is one of the major challenges of current and future research in this area. The ongoing development of high-throughput bioinformatic approaches will further facilitate the identification of conserved structures and the evaluation of their genetic distribution. In addition to novel genetic locations of known ribozymes, new catalytic RNA motifs may be expected to be discovered, as shown recently for the twister, twister sister, pistol, and hatchet ribozymes ^15, 70^. In the area of ribozyme engineering by rational design and *in vitro/in vivo* evolution, exciting results regarding new approaches for the artificial control of gene expression by allosteric ribozymes placed in non-translated regions of transcripts may be anticipated. Also, the search for ribozymes with RNA world relevant activities can be expected to continue with unbroken excitement. In this regard, the catalytic repertoire of XNAzymes ^52, 53^ will certainly be further explored.

Thirty-five years after the discovery of the first catalytic RNA, ribozyme research has not lost the intriguing and highly motivating flair of the first days. There are still many questions to be addressed and much is waiting to be discovered.

## Abbreviations

FANA, 2′-fluoroarabino nucleic acid; SELEX, Systematic evolution of ligands by exponential enrichment; UTR, untranslated region; XNA, xeno nucleic acid. 

## References

[1] Tuerk C Gold L: Systematic evolution of ligands by exponential enrichment: RNA ligands to bacteriophage T4 DNA polymerase. *Science.* 1990;249(4968):505–10. 10.1126/science.2200121 2200121

[2] Ellington AD Szostak JW: *In vitro* selection of RNA molecules that bind specific ligands. *Nature.* 1990;346(6287):818–22. 10.1038/346818a0 1697402

[3] Silverman SK: Deoxyribozymes: selection design and serendipity in the development of DNA catalysts. *Acc Chem Res.* 2009;42(10):1521–31. 10.1021/ar900052y 19572701 PMC2764829

[4] Jaschke A: Artificial ribozymes and deoxyribozymes. *Curr Opin Struct Biol.* 2001;11(3):321–6. 10.1016/S0959-440X(00)00208-6 11406381

[5] Jimenez RM Polanco JA Lupták A: Chemistry and Biology of Self-Cleaving Ribozymes. *Trends Biochem Sci.* 2015;40(11):648–61. 10.1016/j.tibs.2015.09.001 26481500 PMC4630146

[6] Wilson TJ Liu Y Lilley DMJ: Ribozymes and the mechanisms that underlie RNA catalysis. *Front Chem Sci Eng.* 2016;10(2):178–185. 10.1007/s11705-016-1558-2

[7] Liu J Cao Z Lu Y: Functional nucleic acid sensors. *Chem Rev.* 2009;109(5):1948–98. 10.1021/cr030183i 19301873 PMC2681788

[8] Frommer J Appel B Müller S: Ribozymes that can be regulated by external stimuli. *Curr Opin Biotechnol.* 2015;31:35–41. 10.1016/j.copbio.2014.07.009 25146171

[9] Pressman A Blanco C Chen IA: The RNA World as a Model System to Study the Origin of Life. *Curr Biol.* 2015;25(19):R953–63. 10.1016/j.cub.2015.06.016 26439358

[10] Robertson MP Joyce GF: Highly efficient self-replicating RNA enzymes. *Chem Biol.* 2014;21(2):238–45. 10.1016/j.chembiol.2013.12.004 24388759 PMC3943892

[11] Attwater J Wochner A Holliger P: In-ice evolution of RNA polymerase ribozyme activity. *Nat Chem.* 2013;5(12):1011–8. 10.1038/nchem.1781 24256864 PMC3920166

[12] Sczepanski JT Joyce GF: A cross-chiral RNA polymerase ribozyme. *Nature.* 2014;515(7527):440–2. 10.1038/nature13900 25363769 PMC4239201

[13] Hammann C Luptak A Perreault J: The ubiquitous hammerhead ribozyme. *RNA.* 2012;18(5):871–85. 10.1261/rna.031401.111 22454536 PMC3334697

[14] Webb CH Riccitelli NJ Ruminski DJ: Widespread occurrence of self-cleaving ribozymes. *Science.* 2009;326(5955):953. 10.1126/science.1178084 19965505 PMC3159031

[15] Roth A Weinberg Z Chen AG: A widespread self-cleaving ribozyme class is revealed by bioinformatics. *Nat Chem Biol.* 2014;10(1):56–60. 10.1038/nchembio.1386 24240507 PMC3867598

[16] Gupta A Swati D: Hammerhead Ribozymes in Archaeal Genomes: A Computational Hunt. *Interdiscip Sci.* 2016;1–13. 10.1007/s12539-016-0141-3 26758619

[17] Ramesh A Winkler WC: Metabolite-binding ribozymes. *Biochim Biophys Acta.* 2014;1839(10):989–94. 10.1016/j.bbagrm.2014.04.015 24769284

[18] Ward WL Plakos K DeRose VJ: Nucleic acid catalysis: metals, nucleobases, and other cofactors. *Chem Rev.* 2014;114(8):4318–42. 10.1021/cr400476k 24730975 PMC4002065

[19] Hollenstein M: DNA Catalysis: The Chemical Repertoire of DNAzymes. *Molecules.* 2015;20(11):20777–804. 10.3390/molecules201119730 26610449 PMC6332124

[20] Mellin JR Cossart P: Unexpected versatility in bacterial riboswitches. *Trends Genet.* 2015;31(3):150–6. 10.1016/j.tig.2015.01.005 25708284

[21] Soukup GA Breaker RR: Engineering precision RNA molecular switches. *Proc Natl Acad Sci U S A.* 1999;96(7):3584–9. 10.1073/pnas.96.7.3584 10097080 PMC22337

[22] Famulok M Hartig JS Mayer G: Functional aptamers and aptazymes in biotechnology, diagnostics, and therapy. *Chem Rev.* 2007;107(9):3715–43. 10.1021/cr0306743 17715981

[23] Sett A Das S Bora U: Functional nucleic-acid-based sensors for environmental monitoring. *Appl Biochem Biotechnol.* 2014;174(3):1073–91. 10.1007/s12010-014-0990-3 24903959

[24] Townshend B Kennedy AB Xiang JS: High-throughput cellular RNA device engineering. *Nat Methods.* 2015;12(10):989–94. 10.1038/nmeth.3486 26258292 PMC4589471

[25] Rehm C Klauser B Hartig JS: Engineering aptazyme switches for conditional gene expression in mammalian cells utilizing an *in vivo* screening approach. *Methods Mol Biol.* 2015;1316:127–40. 10.1007/978-1-4939-2730-2_11 25967058

[26] Klauser B Atanasov J Siewert LK: Ribozyme-based aminoglycoside switches of gene expression engineered by genetic selection in *S. cerevisiae*. *ACS Synth Biol.* 2015;4(5):516–25. 10.1021/sb500062p 24871672

[27] Nomura Y Zhou L Miu A: Controlling mammalian gene expression by allosteric hepatitis delta virus ribozymes. *ACS Synth Biol.* 2013;2(12):684–9. 10.1021/sb400037a 23697539 PMC3874218

[28] Felletti M Klauser B Hartig JS: Screening of Genetic Switches Based on the Twister Ribozyme Motif. *Methods Mol Biol.* 2016;1380:225–39. 10.1007/978-1-4939-3197-2_19 26552830

[29] Wei KY Chen YY Smolke CD: A yeast-based rapid prototype platform for gene control elements in mammalian cells. *Biotechnol Bioeng.* 2013;110(4):1201–10. 10.1002/bit.24792 23184812

[30] Kennedy AB Vowles JV d'Espaux L: Protein-responsive ribozyme switches in eukaryotic cells. *Nucleic Acids Res.* 2014;42(19):12306–21. 10.1093/nar/gku875 25274734 PMC4231745

[31] Ausländer S Stücheli P Rehm C: A general design strategy for protein-responsive riboswitches in mammalian cells. *Nat Methods.* 2014;11(11):1154–60. 10.1038/nmeth.3136 25282610

[32] Klauser B Hartig JS: An engineered small RNA-mediated genetic switch based on a ribozyme expression platform. *Nucleic Acids Res.* 2013;41(10):5542–52. 10.1093/nar/gkt253 23585277 PMC3664830

[33] Saragliadis A Krajewski SS Rehm C: Thermozymes: Synthetic RNA thermometers based on ribozyme activity. *RNA Biol.* 2013;10(6):1010–6. 10.4161/rna.24482 23595083 PMC4111729

[34] Ketzer P Kaufmann JK Engelhardt S: Artificial riboswitches for gene expression and replication control of DNA and RNA viruses. *Proc Natl Acad Sci U S A.* 2014;111(5):E554–62. 10.1073/pnas.1318563111 24449891 PMC3918795

[35] Mustoe AM Brooks CL Al-Hashimi HM: Hierarchy of RNA functional dynamics. *Annu Rev Biochem.* 2014;83:441–66. 10.1146/annurev-biochem-060713-035524 24606137 PMC4048628

[36] Rehm C Hartig JS: *In vivo* screening for aptazyme-based bacterial riboswitches. *Methods Mol Biol.* 2014;1111:237–49. 10.1007/978-1-62703-755-6_17 24549624

[37] Santoro SW Joyce GF: A general purpose RNA-cleaving DNA enzyme. *Proc Natl Acad Sci U S A.* 1997;94(9):4262–6. 10.1073/pnas.94.9.4262 9113977 PMC20710

[38] Santoro SW Joyce GF: Mechanism and utility of an RNA-cleaving DNA enzyme. *Biochemistry.* 1998;37(38):13330–42. 10.1021/bi9812221 9748341

[39] Zhou C Avins JL Klauser PC: DNA-Catalyzed Amide Hydrolysis. *J Am Chem Soc.* 2016;138(7):2106–9. 10.1021/jacs.5b12647 26854515 PMC4767666

[40] Chandra M Sachdeva A Silverman SK: DNA-catalyzed sequence-specific hydrolysis of DNA. *Nat Chem Biol.* 2009;5(10):718–20. 10.1038/nchembio.201 19684594 PMC2746877

[41] Brandsen BM Hesser AR Castner MA: DNA-catalyzed hydrolysis of esters and aromatic amides. *J Am Chem Soc.* 2013;135(43):16014–7. 10.1021/ja4077233 24127695 PMC3946404

[42] Ponce-Salvatierra A Wawrzyniak-Turek K Steuerwald U: Crystal structure of a DNA catalyst. *Nature.* 2016;529(7585):231–4. 10.1038/nature16471 26735012

[43] Xiang Y Lu Y: DNA as sensors and imaging agents for metal ions. *Inorg Chem.* 2014;53(4):1925–42. 10.1021/ic4019103 24359450 PMC3955431

[44] Zhou Y Tang L Zeng G: Current progress in biosensors for heavy metal ions based on DNAzymes/DNA molecules functionalized nanostructures: A review. *Sens Actuators B Chem.* 2016;223:280–94. 10.1016/j.snb.2015.09.090

[45] Gong L Zhao Z Lv YF: DNAzyme-based biosensors and nanodevices. *Chem Commun (Camb).* 2015;51(6):979–95. 10.1039/c4cc06855f 25336076

[46] Huang PJ Liu M Liu J: Functional nucleic acids for detecting bacteria. *Rev Anal Chem.* 2013;32(1):77–89. 10.1515/revac-2012-0027

[47] Orbach R Willner B Willner I: Catalytic nucleic acids (DNAzymes) as functional units for logic gates and computing circuits: from basic principles to practical applications. *Chem Commun (Camb).* 2015;51(20):4144–60. 10.1039/c4cc09874a 25612298

[48] Karnati HK Yalagala RS Undi R: Therapeutic potential of siRNA and DNAzymes in cancer. *Tumour Biol.* 2014;35(10):9505–21. 10.1007/s13277-014-2477-9 25149153

[49] Robaldo L Berzal-Herranz A Montserrat JM: Activity of core-modified 10–23 DNAzymes against HCV. *ChemMedChem.* 2014;9(9):2172–7. 10.1002/cmdc.201402222 25079672

[50] Cho EA Moloney FJ Cai H: Safety and tolerability of an intratumorally injected DNAzyme, Dz13, in patients with nodular basal-cell carcinoma: a phase 1 first-in-human trial (DISCOVER). *Lancet.* 2013;381(9880):1835–43. 10.1016/S0140-6736(12)62166-7 23660123 PMC3951714

[51] Krug N Hohlfeld JM Kirsten AM: Allergen-induced asthmatic responses modified by a GATA3-specific DNAzyme. *N Engl J Med.* 2015;372(21):1987–95. 10.1056/NEJMoa1411776 25981191

[52] Taylor AI Holliger P: Directed evolution of artificial enzymes (XNAzymes) from diverse repertoires of synthetic genetic polymers. *Nat Protoc.* 2015;10(10):1625–42. 10.1038/nprot.2015.104 26401917

[53] Taylor AI Pinheiro VB Smola MJ: Catalysts from synthetic genetic polymers. *Nature.* 2015;518(7539):427–30. 10.1038/nature13982 25470036 PMC4336857

[54] Pinheiro VB Taylor AI Cozens C: Synthetic genetic polymers capable of heredity and evolution. *Science.* 2012;336(6079):341–4. 10.1126/science.1217622 22517858 PMC3362463

[55] Martin LL Unrau PJ Müller UF: RNA synthesis by *in vitro* selected ribozymes for recreating an RNA world. *Life (Basel).* 2015;5(1):247–68. 10.3390/life5010247 25610978 PMC4390851

[56] Moretti JE Müller UF: A ribozyme that triphosphorylates RNA 5'-hydroxyl groups. *Nucleic Acids Res.* 2014;42(7):4767–78. 10.1093/nar/gkt1405 24452796 PMC3985629

[57] Dolan GF Akoopie A Müller UF: A Faster Triphosphorylation Ribozyme. *PLoS One.* 2015;10(11):e0142559. 10.1371/journal.pone.0142559 26545116 PMC4636267

[58] Ferretti AC Joyce GF: Kinetic properties of an RNA enzyme that undergoes self-sustained exponential amplification. *Biochemistry.* 2013;52(7):1227–35. 10.1021/bi301646n 23384307 PMC3579227

[59] Wochner A Attwater J Coulson A: Ribozyme-catalyzed transcription of an active ribozyme. *Science.* 2011;332(6026):209–12. 10.1126/science.1200752 21474753

[60] Petkovic S Badelt S Block S: Sequence-controlled RNA self-processing: computational design, biochemical analysis, and visualization by AFM. *RNA.* 2015;21(7):1249–60. 10.1261/rna.047670.114 25999318 PMC4478344

[61] Amini ZN Olson KE Müller UF: Spliceozymes: ribozymes that remove introns from pre-mRNAs in trans. *PLoS One.* 2014;9(7):e101932. 10.1371/journal.pone.0101932 25014025 PMC4094466

[62] Hieronymus R Godehard SP Balke D: Hairpin ribozyme mediated RNA recombination. *Chem Commun (Camb).* 2016;52(23):4365–8. 10.1039/c6cc00383d 26923676

[63] Lehman N Vaidya N Yeates JA: RNA-directed recombination of RNA *in vitro*. *Methods Mol Biol.* 2015;1240:27–37. 10.1007/978-1-4939-1896-6_2 25352134

[64] Mutschler H Holliger P: Non-canonical 3'-5' extension of RNA with prebiotically plausible ribonucleoside 2',3'-cyclic phosphates. *J Am Chem Soc.* 2014;136(14):5193–6. 10.1021/ja4127714 24660752 PMC4333585

[65] Sharma AK Plant JJ Rangel AE: Fluorescent RNA labeling using self-alkylating ribozymes. *ACS Chem Biol.* 2014;9(8):1680–4. 10.1021/cb5002119 24896502

[66] Liu Y Wilson TJ McPhee SA: Crystal structure and mechanistic investigation of the twister ribozyme. *Nat Chem Biol.* 2014;10(9):739–44. 10.1038/nchembio.1587 25038788

[67] Ren A Kosutic M Rajashankar KR: In-line alignment and Mg ^2+^ coordination at the cleavage site of the *env22* twister ribozyme. *Nat Commun.* 2014;5: 5534. 10.1038/ncomms6534 25410397 PMC4373348

[68] Eiler D Wang J Steitz TA: Structural basis for the fast self-cleavage reaction catalyzed by the twister ribozyme. *Proc Natl Acad Sci U S A.* 2014;111(36):13028–33. 10.1073/pnas.1414571111 25157168 PMC4246988

[69] Gaines CS York DM: Ribozyme Catalysis with a Twist: Active State of the Twister Ribozyme in Solution Predicted from Molecular Simulation. *J Am Chem Soc.* 2016;138(9):3058–65. 10.1021/jacs.5b12061 26859432 PMC4904722

[70] Weinberg Z Kim PB Chen TH: New classes of self-cleaving ribozymes revealed by comparative genomics analysis. *Nat Chem Biol.* 2015;11(8):606–10. 10.1038/nchembio.1846 26167874 PMC4509812

[71] Harris KA Lünse CE Li S: Biochemical analysis of pistol self-cleaving ribozymes. *RNA.* 2015;21(11):1852–8. 10.1261/rna.052514.115 26385507 PMC4604425

[72] Li S Lunse CE Harris KA: Biochemical analysis of hatchet self-cleaving ribozymes. *RNA.* 2015;21(11):1845–51. 10.1261/rna.052522.115 26385510 PMC4604424

[73] Perreault J Weinberg Z Roth A: Identification of hammerhead ribozymes in all domains of life reveals novel structural variations. *PLoS Comput Biol.* 2011;7(5):e1002031. 10.1371/journal.pcbi.1002031 21573207 PMC3088659

[74] de la Peña M García-Robles I: Intronic hammerhead ribozymes are ultraconserved in the human genome. *EMBO Rep.* 2010;11(9):711–6. 10.1038/embor.2010.100 20651741 PMC2933863

[75] Cervera A De La Peña M: Eukaryotic *penelope*-like retroelements encode hammerhead ribozyme motifs. *Mol Biol Evol.* 2014;31(11):2941–7. 10.1093/molbev/msu232 25135949 PMC4209133

